# Fearful Gaze Cueing: Gaze Direction and Facial Expression Independently Influence Overt Orienting Responses in 12-Month-Olds

**DOI:** 10.1371/journal.pone.0089567

**Published:** 2014-02-20

**Authors:** Reiko Matsunaka, Kazuo Hiraki

**Affiliations:** 1 Graduate School of Arts and Sciences, Department of General System Studies, The University of Tokyo, Tokyo, Japan; 2 CREST, Japan Science and Technology Agency, Saitama, Japan; Birkbeck, University of London, United Kingdom

## Abstract

Gaze direction cues and facial expressions have been shown to influence object processing in infants. For example, infants around 12 months of age utilize others' gaze directions and facial expressions to regulate their own behaviour toward an ambiguous target (i.e., social referencing). However, the mechanism by which social signals influence overt orienting in infants is unclear. The present study examined the effects of static gaze direction cues and facial expressions (neutral vs. fearful) on overt orienting using a gaze-cueing paradigm in 6- and 12-month-old infants. Two experiments were conducted: in Experiment 1, a face with a leftward or rightward gaze direction was used as a cue, and a face with a forward gaze direction was added in Experiment 2. In both experiments, an effect of facial expression was found in 12-month-olds; no effect was found in 6-month-olds. Twelve-month-old infants exhibited more rapid overt orienting in response to fearful expressions than neutral expressions, irrespective of gaze direction. These findings suggest that gaze direction information and facial expressions independently influence overt orienting in infants, and the effect of facial expression emerges earlier than that of static gaze direction. Implications for the development of gaze direction and facial expression processing systems are discussed.

## Introduction

The face provides important signals in human social interaction. Eye gaze direction informs us of stimuli that are salient to others, and facial expressions convey others' mental states. Recent models of face processing have suggested that gaze direction information and facial expressions activate distinct brain modules and are processed in parallel pathways [1]–[3]. Neuroimaging studies have shown that gaze direction and facial expressions activate the common brain regions, such as the superior temporal sulcus (STS) and the amygdala; at the same time, it has been suggested that gaze- and expression-sensitive areas within the regions are dissociable [4] and the timing of gaze and expression processing are different at the early stage of visual processing [5], [6]. It has been further suggested that there are reciprocal projections between these and other brain regions (e.g., the intraparietal sulcus, the orbitofrontal cortex, the fusiform gyrus, and the visual cortex) [7], and several studies have investigated how these social signals affect cognitive processes in infants and adults [8]–[10].

For example, previous studies demonstrated that gaze direction and facial expression influence infants' object processing. By approximately 12 months of age, infants begin to regulate their behaviours when they encounter an ambiguous novel object by monitoring others' social signals; infants are less likely to explore or touch an object when their mothers or experimenters exhibit negative expressions in response to the object [11], [12]. This behaviour is called social referencing. Recent event-related potentials (ERPs) studies have reported an influence of facial expression on infants' object processing. In a gaze-cued target task, infants as young as 3 months old showed an enhanced amplitude of the negative central (Nc) component, considered indicative of attention toward a stimulus, in response to a fearful gaze-cued target relative to a neutral gaze-cued target [13]. Amplitude differences in response to a fearful and a neutral non-gaze-cued target were not observed. These findings indicate that gaze direction and facial expression information influences infants' object processing in an interactive manner.

Object processing first requires attention to that object. Overt orienting, achieved through foveating saccades, toward an object improves the efficiency of object processing [14]. A number of studies have investigated the effects of gaze direction on infants' orienting response; however, the effects of facial expressions have been given little attention. More specifically, it is presently not understood whether these social signals influence infants' overt orienting responses in an interactive or independent manner. As described, the degree to which an infant processes an object depends on the associated social signals. For example, infants exhibit greater engagement with targets that elicit a fearful response in other people. The enhanced attention to fear-evoking objects likely evolved to facilitate the prompt detection of potential danger in the surrounding environment. In the present study, we investigated how gaze direction and facial expression influence overt orienting responses in infants. Monitoring eye movements provides information about real-time orientation toward an object. We therefore monitored infants' eye movements precisely with a high temporal resolution eye-tracker and analysed saccadic reaction time (SRT), saccade amplitude, and saccade velocity: these parameters are commonly assessed in eye-tracking tasks [15]. Given that the neural substrates of the oculomotor system are well understood, detailed eye movement analysis may offer insight into the processing of social signals in infants.

Investigation of the role of gaze direction in attentional orienting toward the side of gaze direction has been previously assessed through the gaze-cueing paradigm [16], [17]. In a standard gaze-cueing paradigm, a schematic or photographic face with neutral expressions and a rightward or leftward gaze is presented centrally as a cue, while the target object is presented laterally, either congruent or incongruent with the gaze direction. The gaze-cueing effect refers to the rapid overt orienting towards a gaze-cued target and is demonstrable in very young infants, and even newborns [18], [19]. This suggests that infants are sensitive to the gaze direction of others and utilize it as an attentional cue; however, it is important to note that, in infants younger than 4 months, the gaze-cueing effect was observed only when there was an apparent perceived motion of an eye gaze shift [18], [20]. As static gaze direction alone produces the gaze-cueing effect in adults [21], it is reasonable to assume that infants develop the enhanced orienting response to *static* gaze direction cues following repeated exposure to them. Previous studies showed that infants are able to discriminate gaze direction in static images; from an early age, infants prefer to look at faces with a direct gaze relative to an averted gaze [22]. Further, when static gaze direction is presented with a target object, it is regarded by infants as a referential cue [23]: although other studies have suggested that infants begin to understand the relationship between looker and object in the second year [24], [25]. Humans frequently encounter situations wherein we must determine the target of others' attention based on static gaze direction alone, as it is impossible to constantly monitor the motion of another person's pupils. While it has been demonstrated that adults rapidly orient their attention towards the gaze direction of others, even in the absence of eye contact [16], few studies have examined the developmental trajectory of the infant orienting response to static gaze direction alone. Therefore, in the present study, we used static gaze direction cues as stimuli and investigated whether these cues facilitate overt orienting responses in older infants.

Several studies have investigated the effect of facial expressions (e.g., happy, sad, disgusted) on infants' overt orienting responses [26], [27]; however, little attention has been given to the effect of fearful expressions [28], despite the ability of such expressions to indicate potential danger. Newborns and infants are able to discriminate some facial expressions (neutral, happy, and fearful expressions) [9], [29]; however, a stable attentional bias to fearful expressions emerges at approximately 7 months of age [30]–[32]. According to behavioural studies in adults, the effects of gaze direction and facial expression on attentional orientation remain controversial. Some evidence suggests an interaction between the processing of gaze direction and facial expression, as the degree of the gaze-cueing effect in these studies was reported to be larger for fearful expressions relative to neutral expressions [33]–[35]. Conversely, reports from other studies suggest gaze direction processing in attentional orientation is independent of facial expression processing, as the magnitude of the gaze-cueing effect was not modulated by expressional type in these studies [36]. Given that infants are highly sensitive to fearful facial expressions, fearful expressions were used in the present study as facial information cues, and it was subsequently examined whether such expressions more effectively stimulate infants' overt orienting responses than do neutral expressions. Further, a critical methodological difference between the present study and previous behavioural studies in adults is that participants responded overtly or manually to the target. Manual reaction time responses are often used to measure orienting speed in adults; however, this method cannot be performed in infants. Therefore, the oculomotor response was used in the present study. Monitoring eye movements is an effective way to measure orienting behaviour in both infant and adult participants.

In the present study, we examined the influence of static gaze direction and facial expression information on the overt orienting responses of 6- and 12-month-old infants in a gaze-cueing paradigm. The results of the present study will indicate either the independence or an interaction of gaze direction and facial expression information in overt orienting responses in infants. If infants' overt orienting responses are modulated by the interaction of gaze direction and facial expression information, infants should regard fearful gaze direction cues as indicative of potential danger, as suggested by previous studies of infant object processing. It follows that infants should exhibit more rapid saccades in response to a fearful gaze-cued target than to a neutral one. Alternatively, if gaze direction information and facial expression information are processed in a parallel manner and independently modulate infants' overt orienting behaviour, analyses should show a main effect of gaze direction and/or facial expression on overt orienting responses. Furthermore, previous studies showed that newborns and infants are able to discriminate some positive and negative facial expressions; however, regarding the fact that infants show a stable attentional bias to fearful expressions in the second half of their first year, we hypothesized that a main effect of facial expression would emerge only in infants older than 7 months.

## Experiment 1

### Method

#### Ethics statement

All parents provided written informed consent. The ethics committee of The University of Tokyo approved the experiment.

#### Participants

Participants were healthy, full-term 6-month (*n* = 16, *M* = 199.4 days, *SD* = 6.3 days; 11 boys, 5 girls) and 12-month-old infants (*n* = 16, *M* = 382.1 days, *SD* = 6.4 days; 7 boys, 9 girls). Ten additional infants were excluded because they did not complete the required number of accepted trials (6-month-olds: *n* = 3; 12-month-olds: *n* = 2) or because of fussiness (6-month-olds: *n* = 3; 12-month-olds: *n* = 2). The criteria for trial acceptance are described in the Data Analysis section. The participants were recruited through birth records at city hall branch offices, and parents who expressed interest in enrolling their children in the study were contacted via email.

#### Apparatus

An infant sat on a parent's lap and faced a 23-inch wide colour monitor (screen resolution 1920×1080 pixels) with an infrared bright pupil eye-tracking system (300 Hz, Tobii TX300, Tobii Technology AB) that sat atop a table. The accuracy of the eye-tracker was 0.5°, on average, for binocular vision. The monitor was about 65 cm away from the infant, and curtains surrounded the table. One camera was set on the table and another camera was set behind the infant. The two images were synchronized with a quad splitter (YH-446C, Mother Tool) to monitor infant behaviour and control the timing of stimulus presentation. Stimulus presentation was controlled by E-Prime software (Psychology Software Tools).

#### Stimuli

The face stimuli were greyscale images of two female models portraying neutral or fearful expressions from the ATR Facial Expression Image database (DB99, ATR-Promotions). Eye gaze direction in these images was manipulated with Adobe Photoshop to create stimuli with leftward and rightward gaze directions. The face pictures were 16.2° in height and 10.8° in width, and the eye region subtended about 2.6° in height and 7.2° in width. Peripheral targets were black and white line drawings of objects subtending a visual angle of 3.3° in height and 5.7° in width, and presented 8.2° to the left or right of the centre of the screen.

#### Procedure

**Figure 1:**
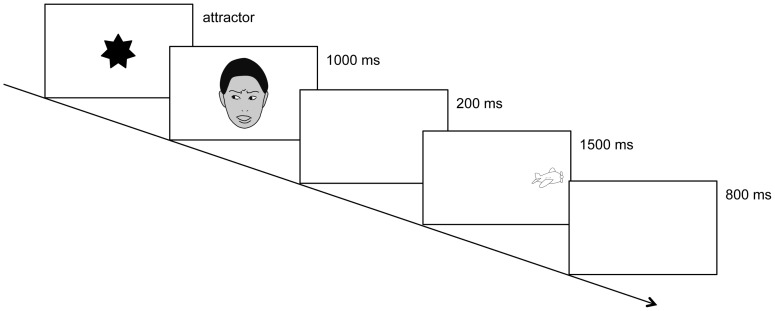
Example of a stimulus presentation sequence. The expression is fearful; the target appears on the incongruent side. At the beginning of each trial, an attractor stimulus was presented on the monitor until the infant fixated on the object. Then a face with a leftward or rightward gaze direction showing a neutral or fearful expression was presented for 1000 ms. The monitor then displayed a blank screen for 200 ms, followed by the presentation of a leftward or rightward peripheral target for 1500 ms. Another blank screen was then presented for 800 ms, and the next trial started. The images of faces shown here do not depict the actual stimuli, but are intended only as examples.

A five-point calibration procedure was repeated until more than three calibration points for each eye were successful (Tobii Studio, Tobii Technology AB). The experiment began after successful calibration. Figure 1 shows the sequence of events within a trial. At the beginning of each trial, an attractor stimulus, a colourful object that shrank and expanded to about 5° in width and 5° in height at the maximum, was presented on the monitor until the infant fixated on the object. When the experimenter judged that the infant was fixating, a trial was initiated. A face with a leftward or rightward gaze direction showing a neutral or fearful expression was presented for 1000 ms. The monitor then displayed a blank screen for 200 ms, followed by the presentation of a leftward or rightward peripheral target for 1500 ms. Therefore, the duration of the stimulus onset asynchrony (SOA) was 1200 ms: this duration was selected to maintain consistency with the SOA used for infants in similar studies (i.e., an SOA duration greater than 1000 ms) [18]–[20]. Another blank screen was then presented for 800 ms, and the next trial then began. All stimuli were presented on a uniform white background. A pseudo-random trial sequencing approach was used wherein the target appeared equally often on the right or left side, with the restriction that the target did not appear on the same side for more than three consecutive trials. Gaze direction and facial expression were independently varied. Presentation order was counterbalanced in blocks of 16 trials, with a trial in each condition presented four times in a block. Calibration and drift correction of the position signal were repeated every 16 trials to ensure accuracy during the experiment. The experiment continued until the infant completed three blocks, or became fussy or inattentive.

#### Data analysis

During each trial, a graph of recorded horizontal gaze position and velocity data versus time was generated during target presentation. Velocity data were generated by differentiating gaze point data with respect to time. Saccades were identified manually [37], and the onset of a saccade was defined as an eye movement with a velocity greater than 15°/s [38]. Similarly, the end of a saccade was defined as a gaze position that fell within an area of 0.5° for more than 10 consecutive points of gaze data (about 35 ms). After a saccade was determined, SRT was calculated by subtracting the target onset time from the saccade onset time. A trial was excluded if the SRT was three standard deviations above the overall mean value; if there were any missing data points due to excessive movement or blinking during the defined saccade; or if the total face-orientation time was less than 500 ms. For accepted trials in each condition, we calculated saccade amplitude in degrees and peak velocity. The saccade amplitude and peak velocity were calculated for all included trials in each condition. Amplitude and peak velocity measures of saccades provide an indication of the efficacy of the saccadic burst generator in the brainstem, and are often used to check the maturation of the brainstem saccade-related regions in developmental studies [15]. For both age groups, all measures were analysed with a repeated-measures analysis of variance (ANOVA), with facial expression (neutral and fearful) and gaze congruency (congruent and incongruent) as within-subjects factors. Additionally, we analysed participants' viewing patterns during the face cue presentation. We created two areas of interest (AOIs) on the face: one around the eyes and another around the mouth. The eye AOI spanned above the eyebrows through the bridge of the nose, and the mouth AOI consisted of the mouth [39]. We calculated the total looking duration for each AOI.

### Results

Data from participants with at least two accepted trials in each condition were included in the final analysis. Infants completed an average of 44 trials (range: 33–48 trials, *SD* = 4.8). Trials were rejected for excessive movements or blinks (28%), or if the SRT was three standard deviations above the mean (6.0%), as described earlier. There were no anticipatory eye movements (SRT below 80 ms) or saccades in the wrong direction. The average number of accepted trials in each condition for both age groups is summarized in Table 1. The number of accepted trials did not differ between conditions.

**Table 1:** Mean Scores of Analysed Variables for Each Condition in 6- and 12-month-olds in Experiment 1.

	Neutral				Fearful			
Analysed Variables	Congruent		Incongruent		Congruent		Incongruent	
6-month-olds								
Accepted trials	7.0	(2.7)	7.5	(2.4)	7.3	(2.5)	7.1	(1.9)
Saccadic reaction time [ms]	206.6	(26.0)	211.5	(20.9)	214.6	(27.6)	210.8	(27.3)
Saccade Amplitude [°]	9.2	(0.9)	9.2	(0.8)	9.1	(0.7)	9.2	(0.6)
Peak Velocity [°/s]	403.6	(69.7)	407.8	(65.8)	400.0	(59.8)	402.5	(62.2)
12-month-olds								
Accepted trials	7.3	(2.6)	6.8	(2.6)	6.9	(2.5)	7.4	(2.5)
Saccadic reaction time [ms]	187.4	(26.4)	188.9	(18.3)	181.2	(14.5)	175.3	(15.6)
Saccade Amplitude [°]	8.8	(0.8)	9.3	(0.7)	9.2	(0.8)	9.3	(0.8)
Peak Velocity [°/s]	429.8	(76.2)	424.4	(54.3)	440.6	(68.7)	429.9	(67.2)

*Note*. Values in parentheses are standard deviations.

#### Saccadic reaction time (SRT)

The mean SRT for each condition is shown in Table 1. For the 6-month-olds, the ANOVA showed no main or interaction effects. For the 12-month-olds, significantly faster SRTs were observed in the fearful expression condition (178.3 ms) relative to the neutral expression condition (188.1 ms) (*F* (1, 15)  = 6.45, *p* = .023, η_p_^2^ = 0.30). There were no significant main or interaction effects of gaze congruency.

#### Saccade amplitude

Saccade amplitudes for all conditions are shown in Table 1. There were no main or interaction effects of saccade amplitude observed in the 6-month-olds. In the 12-month-olds, there was a marginally significant main effect of gaze congruency (*F* (1, 15)  = 4.01, *p* = .06, η_p_^2^ = 0.21), wherein amplitudes were greater during incongruent (9.3°) than congruent trials (9.0°). The average saccade duration and initial eye position at saccade onset did not differ between conditions.

#### Peak velocity

Mean saccade peak velocity for each condition is shown in Table 1. There were no main or interaction effects of peak velocity observed in either age group.

#### Total looking duration

**Figure 2:**
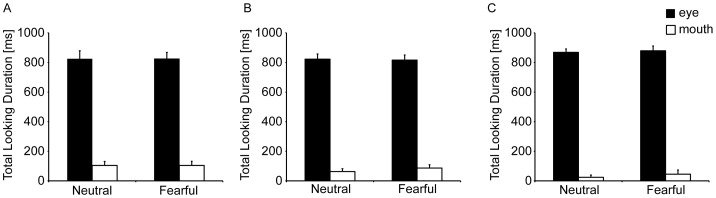
Mean total looking duration for the eye and mouth regions for neutral and fearful expressions. (A) 6-month-olds (B) 12-month-olds in Experiment 1 (C) 12-month-olds in Experiment 2. Error bars indicate standard errors of the mean.

Total looking durations towards each AOI for all conditions are shown in Figure 2A (6-month-olds) and 2B (12-month-olds). For both age groups, a repeated-measures ANOVA, with facial expression (neutral and fearful) and AOI (eyes, mouth) as within-subject factors, showed a significant main effect of AOI. Infants looked longer at the eye region than the mouth region (6-month-olds: *F* (1, 15)  = 94.4, *p*<.01, η_p_^2^ = 0.86; 12-month-olds: *F* (1, 15)  = 202.8, *p*<.01, η_p_^2^ = 0.93).

### Discussion

Results showed that 12-month-olds exhibited more rapid orientation toward the peripheral target when a fearful expression was presented as a central cue than when a neutral expression was presented, irrespective of gaze congruency. Conversely, no such effect of fearful expression was observed in 6-month-olds. The effect of fearful expressions on SRTs is consistent with some previous studies in adults. For example, Graham et al. (2010) [33] reported that manual RT to a target is shorter in response to a fearful face than a neutral face. In addition, recent research has shown that fearful expressions modulate and enhance the early stage of visual processing [40], [41]. It is consequently possible that the presentation of fearful expressions enhanced the 12-month-olds' visual processing and enabled faster detection of the peripheral target. Furthermore, the lack of effect of fearful expressions on SRTs in 6-month-olds in the present study is consistent with previous reports that infants exhibit stable sensitivity to fearful expressions at approximately 7 months old [32]. Further, the failure of fearful expressions to significantly alter responses in 6-month-olds suggests that the faster SRTs associated with fearful expression presentation in 12-month-olds may not reflect a simple orienting response triggered by salient stimuli. For example, it may not be due to low-level feature differences between neutral and fearful expressions (i.e., widely open eyes or mouth). Both 6- and 12-month-old infants looked longer at the eye region relative to at the mouth region, irrespective of facial expressions, in the present study.

A marginal effect of gaze congruency on saccade amplitude in 12-month-olds suggests that the overt orienting system in 12-month-olds is partially affected by static gaze direction cues. Although this finding is difficult to interpret due to minimal reports on the measurement methodology of saccade dynamics, except for that for SRT analysis within the gaze-cueing paradigm; the orientation of 12-month-olds in response to static gaze direction may have resulted from an ability to predict the side in which the target would appear [42].

The results of Experiment 1 suggest that SRTs in 12-month-old infants are influenced by fearful expressions, and gaze direction information and facial expression cues independently influence overt orienting responses. In Experiment 2, we included as a stimulus a face with a forward gaze direction as a cue (i.e. forward-gaze condition), and further investigated the effect of static gaze direction and facial expression on overt orienting responses in 12-month-olds. Unlike the averted-gaze face, the forward-gaze face did not have a directional cue. If 12-month-olds' overt orienting is stably influenced by fearful expressions, as indicated in Experiment 1, results should indicate a main effect of facial expression on SRTs, even in a forward-gaze condition. Alternatively, if 12-month-olds' overt orienting is influenced by the directional component of the presented eye gaze, results should show a main effect of gaze direction (rightward, leftward, and forward) on SRTs: infants would exhibit more rapid saccades when a face with a rightward or leftward gaze direction is presented as a cue than when a face with a forward gaze direction is presented.

## Experiment 2

In Experiment 2, we added a forward-gaze condition (face with a forward gaze direction) and examined whether the effect of fearful expressions on SRTs observed in Experiment 1 could be replicated.

### Method

#### Ethics statement

All parents provided written informed consent. The ethics committee of The University of Tokyo approved the experiment.

#### Participants

Sixteen healthy, full-term 12-month-old infants (*M* = 383.6 days, *SD* = 4.8 days; 8 boys, 8 girls) participated in Experiment 2. An additional nine infants were excluded because of fussiness (*n* = 5) or an inadequate number of accepted trials (*n* = 4). Recruitment was performed as described in Experiment 1. None of the infants had participated in Experiment 1.

#### Apparatus, stimuli, and procedure

The apparatus, stimuli, and procedure were the same as in Experiment 1, except for the inclusion of forward-gazing faces as stimuli.

#### Design

The experimental design was the same as in Experiment 1, except for the addition of a forward-gaze condition. In this condition, the model's gaze direction was straight ahead, and the target appeared equally often on the left or right side of the face. Thus, there were three gaze-congruency conditions: congruent, incongruent, and forward-gaze. The presentation order was counterbalanced in blocks of 12 trials, with a trial in each condition presented two times in a block. The experiment continued until the infant became fussy or inattentive, or completed 4 blocks.

#### Data analysis

As in Experiment 1, we analysed SRT, saccade amplitude, peak velocity, and total looking duration at the eyes and the mouth regions. All variables, except for total looking duration, were analysed by a repeated-measures ANOVA, with facial expression (neutral and fearful) and gaze congruency (congruent, incongruent, and forward-gaze) as within-subjects factors.

### Results

Data for 16 participants with at least two accepted trials in each condition were included in the final analysis. The infants completed an average of 42 trials (range: 32–48 trials, *SD* = 6.1 trials). Trials were rejected for excessive movements or blinks (39%), or if the average SRT was three standard deviations above the overall mean (2.1%). There were no anticipatory eye movements or saccades in the wrong direction. The average number of accepted trials in each condition is summarized in Table 2. Analysis of the number of accepted trials did not yield significant effects.

**Table 2:** Mean Scores of Analysed Variables for Each Condition in Experiment 2.

	Neutral						Fearful					
Analysed Variables	Congruent		Incongruent		Forward-gaze		Congruent		Incongruent		Forward-gaze	
Accepted trials	4.3	(1.6)	3.7	(1.3)	4.3	(1.5)	4.3	(1.7)	4.3	(1.6)	4.5	(1.5)
Saccadic reaction times [ms]	171.7	(21.4)	175.6	(20.0)	177.6	(22.0)	167.4	(14.4)	161.3	(18.2)	171.0	(18.1)
Saccade Amplitude [°]	8.7	(0.9)	8.8	(0.9)	9.2	(1.0)	9.1	(0.8)	9.1	(0.7)	9.1	(0.7)
Peak Velocity [°/s]	372.0	(35.7)	381.6	(49.3)	385.1	(38.3)	373.4	(33.9)	378.3	(42.5)	391.1	(34.1)

*Note*. Values in parentheses are standard deviations.

#### SRT

**Figure 3:**
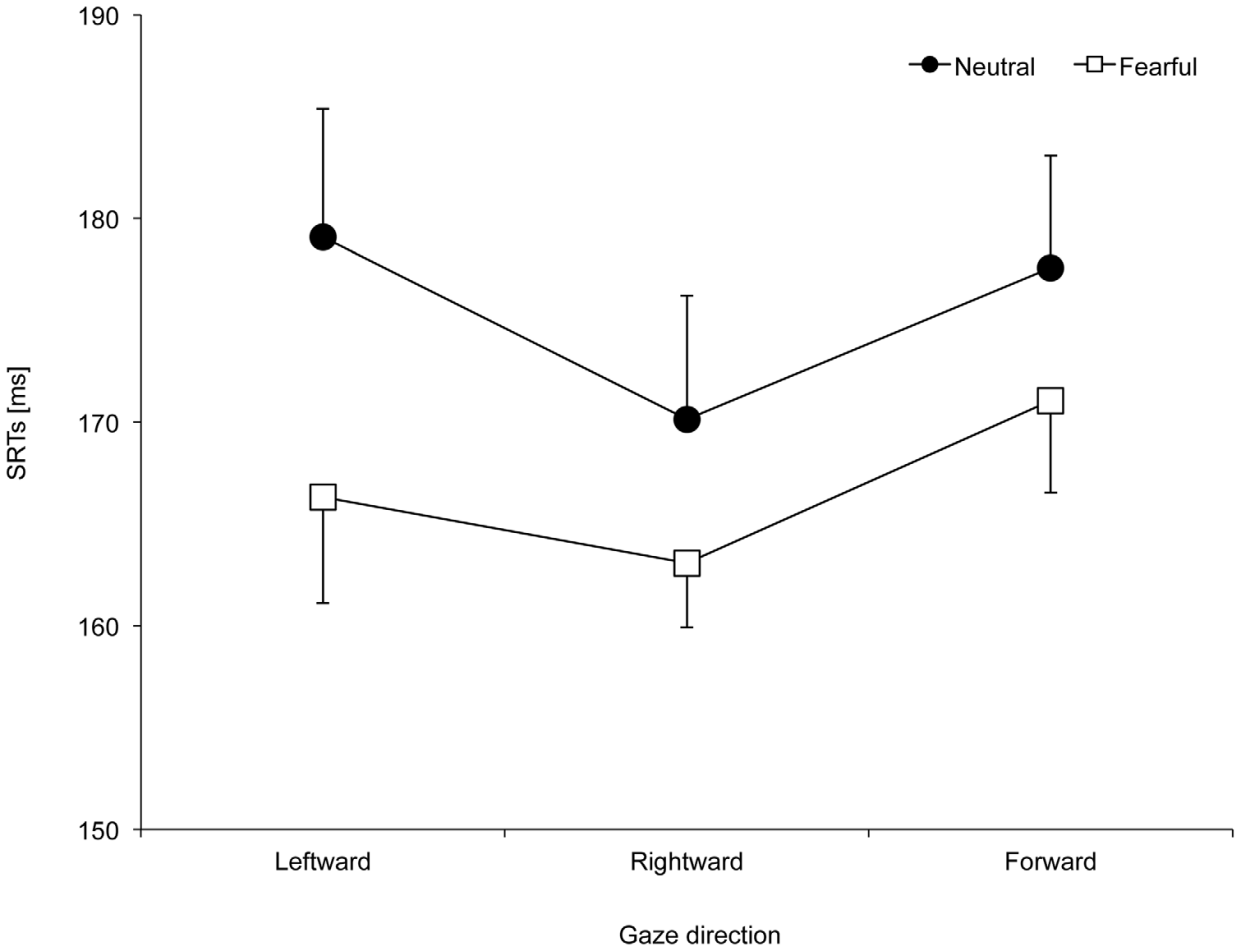
Mean saccadic reaction times (SRTs) for gaze direction conditions among neutral and fearful expressions. SRTs for leftward, rightward, and forward gaze direction conditions for neutral and fearful expressions in Experiment 2 are shown. Error bars indicate standard errors of the mean.

The SRT means are shown in Table 2. There was a significant main effect of facial expression (*F* (1, 15)  = 6.84, *p* = .020, η_p_^2^ = 0.31), with faster SRTs observed in the fearful expression condition (166.6 ms) relative to the neutral expression condition (175.0 ms). No significant main or interaction effects of gaze congruency were observed. Furthermore, to examine whether eye gaze direction (averted or straight) affected overt orienting responses, a repeated-measures ANOVA was performed, with facial expression (neutral and fearful) and eye gaze direction (rightward, leftward, and forward) as within-subjects factors. Previous research has demonstrated that the disengagement of attention from a face is modulated by gaze direction [43]: a face with a forward gaze delays the disengagement of attention relative to a face with an averted gaze. Results showed a significant main effect of facial expression, with faster SRTs observed in the fearful expression condition (166.8 ms) than in the neutral expression condition (175.6 ms) (*F* (1, 15)  = 6.72, *p* = .020, η_p_^2^ = 0.31), as shown in Figure 3. There were no significant main or interaction effects of eye gaze direction.

#### Saccade amplitude

The mean saccade amplitude for each condition is shown in Table 2. There were no significant effects of saccade amplitude. Saccade duration and the initial position at saccade onset were not significantly different between conditions.

#### Peak velocity

The means of the saccade peak velocities are shown in Table 2. No significant effects of peak velocity were observed.

#### Total looking duration

Total looking durations at each AOI for all conditions are shown in Figure 2C. A repeated-measures ANOVA, with facial expression (neutral and fearful) and AOI (eyes, mouth) as within-subject factors, showed a significant main effect of AOI. Infants looked longer at the eye region than at the mouth region (*F* (1, 15)  = 349.2, *p*<.01, η_p_^2^ = 0.96).

### Discussion

Consistent with the results of Experiment 1, 12-month-olds exhibited more rapid overt orienting in response to a fearful expression than a neutral expression, irrespective of gaze congruency and gaze direction. However, unlike Experiment 1, there was no effect of gaze congruency on saccade amplitude. This unstable effect of static gaze direction cues on overt orienting suggests that the gaze-triggered, attentional orienting system is still developing at 12 months of age.

With regard to SRTs, infants quickly exhibited saccades toward the target when a fearful expression was presented, irrespective of gaze congruency. Additionally, eye gaze direction did not influence overt orienting in 12-month-olds. We did not observe SRT differences in response to faces with an averted (rightward or leftward) or forward gaze. Previous studies report that infants exhibit delayed disengagement from faces with fearful expressions relative to those with neutral expressions [44] when the face stimuli were still visible at target onset (overlap paradigm). The lack of delayed disengagement from fearful expressions in the present study might have resulted from the difference in the time course of procedure: the face stimuli in the present study had already disappeared at the time of target onset (gap paradigm). Infants did not need to disengage their attention from the face, and could rapidly respond to the target after the fearful expression was presented. This result suggests that fearful expressions have greater influence on the overt orienting system than static gaze direction cues in 12-month-old infants.

## General Discussion

The present study investigated how gaze direction and facial expression influence 6- and 12-month-old infants' overt orienting responses in the gaze-cueing paradigm. Results showed an effect of facial expression on SRTs in 12-month-olds: saccades toward a target with a fearful expression were faster than those toward a target with a neutral expression, irrespective of gaze direction. In 6-month-olds, no effects of facial expression and static gaze direction cues were found. Regarding the development of gaze direction and facial expression processing, these results suggest that gaze direction and facial expression independently affect overt orienting in infants, at least in response to fearful expressions. Further, the gaze-triggered attention system does not yet function in an adult-like manner within the first year of life.

The present results suggest that gaze direction and facial expression are processed in parallel and independently influence overt orienting responses at 12 months of age. This view is consistent with models of face processing [1]–[3] that propose gaze direction and facial expression are processed in parallel, independent pathways. It is unclear how these parallel, independent pathways are tuned during development; however, with regard to the processing of facial expressions, the absence of a distinct orienting response to fearful expressions in 6-month-olds is consistent with previous findings that infants demonstrate the stable attentional bias toward fearful facial expressions around 7 months of age [30]–[32]. Further, the lack of effect of fearful expressions in 6-month-olds also suggests that the shorter SRT in response to fearful expressions observed in 12-month-olds was not simply due to the perceptual/low-level image differences between fearful and neutral expressions, such as enlarged eyes or the opened mouth, because both 6- and 12-month-olds looked longer at the eye region than at the mouth region, irrespective of the emotion of presented face stimuli. Rather, evidence suggests 12-month-olds may detect the informational value conveyed by fearful expressions.

Recent neuroimaging studies have shown that a fearful expression elicits enhanced activity in the amygdala and visual cortex [40], [45]. It has also been suggested that the emotion-related subcortical route (including the amygdala) is functional from early infancy [46]. Thus, it is assumed that gaze direction and facial expression are initially processed in parallel, independent pathways [5], [6], and the presentation of fearful expressions lowered the detection threshold of peripheral targets. This could have led to the rapid overt orienting responses toward the target observed in 12-month-olds in the present study.

Face stimuli in the present study had already disappeared at the time of target onset (gap paradigm), so infants did not need to disengage their attention from the face. It is reasonable to suggest that fearful expressions affect 12-month-olds' orienting of attention, but not disengagement of attention. The present findings suggest that sensitivity to static gaze direction cues is still immature at the first year of life; thus, rapid target orientation in response to a fearful expression, irrespective of eye gaze direction, is advantageous because it enables the timely assessment of potential danger. Whether the effect of facial expression is specific to fearful expressions or induced by other expressions requires further investigation.

The absence of a gaze-cueing effect in the present study suggests that static gaze direction cues do not yet stimulate overt orienting responses at 12 months of age. The lack of a gaze-cueing effect may have resulted from the duration of SOA. Several previous studies in adults demonstrated that the magnitude of cueing effects with manual key press responses varied according to the duration of the SOA (e.g., [16]). In the present study, we followed the previous infant experimental setting of SOA (i.e., an SOA duration greater than 1000 ms) [18]–[20]; however, a different SOA might induce the gaze-cueing effect in infants. Further research is needed to test this possibility.

A second possible explanation for the lack of a gaze-cueing effect is the immaturity of frontal lobe processes in infants. Recent research suggests that the frontal lobe plays a key role in gaze-triggered attentional orientation [47], [48]. For example, Vecera and Rizzo (2006) [48] reported that a participant suffering from frontal-lobe damage demonstrated significant cueing effects to peripheral cues, but not centrally presented gaze direction cues. This finding suggests that gaze-triggered attentional shifts are mediated by a frontal lobe process. Further, other studies have demonstrated that the frontal lobe process that mediates saccade planning functions differently in 12-month-olds than in adults [49]. Indeed, evidence suggests the frontal lobe is still developing during preschool years [50]. Taken together, these findings support the notion that frontal lobe immaturity may have contributed to the absence of a gaze-cueing effect, especially for *static* gaze direction cues, in infants younger than 12 months of age. The relationship between the gaze-triggered attentional shift and frontal lobe development may be elucidated through direct measures of brain activity, such as event-related potentials recording.

A third possible explanation is the lack of eye contact engagement prior to orienting. Eye contact is one of the most common communicative signals used by humans, and infants are very sensitive to this signal (e.g., [51]). In the present study, we investigated the effect of static gaze direction alone on infants' overt orienting responses; since the faces with an averted gaze were presented first, there were no eye contact engagement phases. Inclusion of an eye contact engagement phase might elicit a perceived eye gaze shift, and consequently produce the gaze-cueing effect, as previously reported [18]–[20]. Given the previous findings that static gaze direction alone without prior eye contact produces the gaze-cueing effect in adults, it is probable that the sensitivity to static gaze direction increases during development. Future studies should examine whether eye contact prior to orienting produces the gaze-cueing effect in infants around 12 months of age.

The present study analysed SRTs, saccade amplitude, and peak velocity, which are common measures of eye movement in saccade tasks (e.g., gap/overlap, anti-saccade, and memory-guided saccade tasks) [15]. We found a marginal effect of gaze congruency on saccade amplitude; however, the effect was limited to Experiment 1. The results suggest that the gaze-triggered attention system is still immature and unstable in 12-month-old infants. As described above, this result may be related to frontal lobe immaturity in 12-month-olds. It has been previously reported that adult participants showed the gaze-cueing effect in overt orienting responses when a neutral static averted gaze face was presented as a cue; however parameters such as amplitude, velocity, and duration have not been examined [21], [52]. Analysis of eye movement attributes, such as amplitude and peak velocity, using the gaze-cueing paradigm in adults, could potentially further the interpretation of the present findings in 12-month-olds.

In conclusion, our results show that the effect of facial expression on infants' overt orienting responses emerges earlier than that of static gaze direction, and these signals independently influence the response, at least when fearful expressions were used as stimuli. Specifically, the presentation of fearful expressions shortened the time required for 12-month-olds to begin overt orientation toward the peripheral target. The early influence of facial expression likely results from the accelerated development of functions mediated by emotion-related subcortical systems (e.g., the amygdala) relative to the gaze-triggered attentional orienting systems (e.g., frontal lobes). With respect to gaze direction and facial expression processing pathways for overt orienting responses, we hypothesized two potential routes: one is a fast and coarse subcortical pathway (including the amygdala, the superior colliculus, and the pulvinar) that mainly processes emotion-related information and induces rapid overt orienting; and the other is a slower, fine cortical pathway (including the superior temporal sulcus and the dorsal and ventral fronto–parietal attention networks) that processes gaze direction information and induces gaze-triggered attentional orienting responses. The neural substrates of the oculomotor system are well understood; however, the pathway through which social signals, such as gaze direction information and facial expression information, influence saccade response remains unclear. The manner in which social signals influence the distributed neural network is an interesting topic for future research.
